# Alcohol and Circadian Disruption Minimally Impact Bone Properties in Two Cohorts of Male Mice While Between‐Cohort Differences Predominate: Association With Season of Birth?

**DOI:** 10.1002/jbm4.10591

**Published:** 2022-01-13

**Authors:** Brittany M Wilson, Brittany R Witkiewics, Robin M Voigt, Christopher B Forysth, Ali Keshavarzian, Frank C Ko, Amarjit S Virdi, D Rick Sumner

**Affiliations:** ^1^ Department of Anatomy and Cell Biology Rush University Medical Center Chicago IL USA; ^2^ Department of Orthopedic Surgery Rush University Medical Center Chicago IL USA; ^3^ Department of Internal Medicine Rush University Medical Center Chicago IL USA; ^4^ Center for Integrated Microbiome and Chronobiology Research Rush University Medical Center Chicago IL USA

**Keywords:** ALCOHOL, BONE, CIRCADIAN DISRUPTION, CIRCANNUAL, MICRO‐COMPUTED TOMOGRAPHY

## Abstract

Many lifestyle factors affect bone. Sleep deprivation increases risk for fractures and alcohol consumption can lead to alterations in the skeleton. How combined exposure to these two risk factors affects bone is unclear. Thus, we sought to determine the effects of circadian rhythm disruption and chronic alcohol intake on bone structure and mechanical properties in mice. A total of 120 male C57BL/6J mice were used in two cohorts of 60 mice each because of limited availability of light‐tight housing cabinets. One cohort was born in winter and the other in summer. Mice were randomly assigned to circadian disruption (weekly shifting of the light/dark cycle) and control (no shifting) groups beginning at 8 to 12 weeks of age for 12 weeks at which time mice were administered an alcohol‐containing or control diet for an additional 10 weeks. Bone structure and mechanical properties of the femur were assessed by micro‐computed tomography and three‐point bending, respectively. The initial data analysis revealed a likely cohort effect. Thus, we used a three‐way analysis of variance to assess the effects of circadian rhythm disruption, alcohol intake, and cohort. Circadian rhythm disruption alone had minimal effects on bone structure and mechanical properties. Alcohol intake reduced body mass and had minimal effects on cortical bone regardless of circadian disruption. Alcohol intake resulted in higher trabecular bone volume, but these beneficial effects were blunted when circadian rhythm was disrupted. Cohort significantly affected body size, many cortical bone structure outcomes, some trabecular bone structure outcomes, and tissue‐level material properties. Thus, cohort had the predominant effect on bone structure and mechanical properties in this study, with chronic alcohol intake and environmental circadian disruption having less consistent effects. The data indicate that season of birth may affect skeletal phenotypes and that studies requiring multiple cohorts should determine if a cohort effect exists. © 2021 The Authors. *JBMR Plus* published by Wiley Periodicals LLC on behalf of American Society for Bone and Mineral Research.

## Graphical abstract

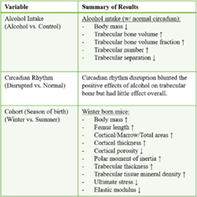

## 1 Introduction

Many lifestyle factors such as sleep patterns and diet influence bone. Understanding these factors may help clarify risk for osteopenia or fracture as well as identify strategies to maintain bone mass and strength. In this study, we sought to determine if disruption of circadian rhythm and chronic alcohol consumption individually or in combination affected bone.

Biological rhythms are repetitive patterns in physiological processes and behavior that recur with predictable frequency. For instance, circadian rhythms cycle daily and circannual (or seasonal) rhythms cycle yearly. A master circadian clock (located in the suprachiasmatic nucleus) and a master seasonal pacemaker (in the pituitary gland) direct these biological rhythms and are robustly influenced by exposure to light (ie, light at night) and light/dark cycles (eg, changes in length of day with season).^(^ ^1^, ^2^, ^3^ ^)^

Circadian rhythms are driven by transcriptional‐translational feedback loops (ie, core clock elements) that oscillate over a 24‐hour period and are found in nearly every cell in the mammalian body, including bone cells.^(^ ^4^ ^)^ Genetic disruption of the master or local bone clock leads to skeletal abnormalities in mice.^(^ ^5^, ^6^ ^)^ The circadian clock can also be disrupted environmentally by altering light/dark cycles as occurs in individuals engaged in shift work (eg, working overnight or rotating shift). Several studies demonstrate that reduced bone mineral density and increased fracture risk are associated with environmental circadian rhythm disruption in humans,^(^ ^7^, ^8^, ^9^ ^)^ whereas two studies report minimal, inconsistent impact of circadian rhythm disruption on murine bone structure with effects observed in other bone‐related parameters.^(^ ^10^, ^11^ ^)^

Alcohol is widely consumed by humans and can exert complex effects on the skeleton depending on myriad factors, including age and pattern of use.^(^ ^12^ ^)^ For instance, low to moderate alcohol consumption in adults may be protective due to a reduced bone remodeling rate and thus reduced age‐related bone turnover and loss.^(^ ^12^ ^)^ However, binge drinking and consumption of alcohol by skeletally immature individuals may lead to deleterious effects including reduction in bone mineral density and increased fracture risk.^(^ ^13^, ^14^ ^)^

There is much to be learned about how combining these factors impacts bone. For example, when combined with circadian rhythm disruption, it is possible that chronic alcohol intake could adversely affect bone, even in skeletally mature individuals. Thus, we tested the hypothesis that environmental circadian disruption and chronic alcohol intake affect bone structure and mechanical properties.

## 2 Materials and Methods

### 2.1 Study design

All procedures used in this study were approved by the Institutional Animal Care and Use Committee at Rush University Medical Center (RUMC). Mice were wild‐type C57BL/6J males (*n* = 120; Jackson Laboratory, Bar Harbor, ME, USA). Mice were housed individually within ventilated, light‐tight cabinets to allow for manipulation of light cycles independent of the ambient light cycles with pure corn cob bedding (Harlan, #7097) and were fed standard rodent chow (Harlan‐Teklad [currently Envigo‐Teklad, Indianapolis, IN, USA] Global 18% Protein Rodent Diet, product #2018) and water both *ad libitum*, except as noted below. Mice were housed and maintained by the Center for Circadian Rhythms and Alcohol‐Induced Tissue Damage at RUMC and are a subset of animals from a previously published study.^(^ ^15^ ^)^ Because of a limited number of light‐tight housing units, two different cohorts of mice, one born in the winter and the second in the summer, were acquired from the same room at Jackson Laboratory (JaxEast: MP15). Temperature and humidity were monitored throughout the study and were maintained at 22 ± 2°C and 20% to 70%, respectively. Temperature and humidity were not different over time between cohorts.

Mice were 8 to 12 weeks of age at the start of the experiment and were randomly assigned to one of four treatment groups (*n* = 15/group) within each of two cohorts. Within each cohort, the groups were: (i) circadian normal, control diet; (ii) circadian normal, alcohol diet; (iii) circadian disrupted, control diet; and (iv) circadian disrupted, alcohol diet. The circadian normal mice were subjected to consistent light/dark cycles (lights on at 7 a.m., lights off at 7 p.m.), whereas the circadian‐disrupted mice were subjected to weekly alterations in light/dark cycles (ie, 12‐hour light/dark inversions: week 1 light/dark; week 2 dark/light; week 3 light/dark, etc.) for 22 weeks.

During the last 10 weeks of the study, mice were transitioned from standard rodent chow to a liquid diet to begin alcohol treatment.^(^ ^16^ ^)^ This transition included a 2‐week gradual increase in alcohol dose followed by 8 weeks on the full chronic alcohol concentration in the alcohol groups (29% of total calories, 4.5% v/v; additional dietary information in Appendix S1). Control mice were fed an isocaloric diet in which alcohol calories were replaced with dextrose. Food was prepared fresh daily and served in graduated sipper tubes.

At study termination, mice (30 to 34 weeks old) were euthanized by conscious decapitation. In each group, mice were euthanized every 8 hours (*n* = 5 per time) to control for time‐of‐day effects in the parent study;^(^ ^15^ ^)^ however, since time of the day had negligible effects on bone outcomes, it was not further considered (Appendix S1). At necropsy, body weight was recorded, serum was collected for blood alcohol measurement (reported in Appendix S1), and the right femur was isolated, cleaned of soft tissue, and stored in saline‐soaked gauze at −20°C until analysis.

### 2.2 Micro‐computed tomography (μCT)

Femora were brought to room temperature and scanned (Scanco μCT40; Scanco Medical, Brüttisellen, Switzerland) parallel to the long axis of the bone while submerged in phosphate buffered saline. Scans were completed using 55 kV, 145 μA, 300 ms integration time, and a voxel size of 10 μm. A hydroxyapatite phantom was scanned weekly to monitor calibration of the X‐ray source of the machine over time. Femur length was measured using the scout view.

The cortical bone analysis region of interest (ROI) spanned from the periosteal to endocortical surfaces and included 1 mm centered at the midshaft of the femur. A threshold of 300 Scanco units was used to identify bone. In accordance with the ASBMR guidelines for the use of μCT in rodents,^(^ ^17^ ^)^ reported outputs include total cross‐sectional area (mm^2^), cortical bone area (mm^2^), marrow area (mm^2^), cortical bone fraction (bone area/total cross‐sectional area × 100), cortical thickness (mm), cortical porosity (%), cortical tissue mineral density (mg hydroxy apatite [HA]/cm^3^), and polar moment of inertia (mm^4^).

The trabecular bone analysis ROI included the entire medullary area deep to the endocortical surface, beginning sufficiently superior to the distal femoral growth plate to avoid the primary spongiosa, and continued proximally to 30% of the length of the bone. On average, the ROI was 2.68 ± 0.7 mm long. The threshold for bone was set to 250 Scanco units. Trabecular outputs include total volume (mm^3^), bone volume (mm^3^), bone volume fraction (bone volume/total volume × 100), trabecular number (1/mm), trabecular thickness (μm), trabecular separation (μm), and trabecular tissue mineral density (mg HA/cm^3^).

### 2.3 Whole‐bone mechanical testing

A three‐point bend test was used to assess mechanical properties of the femur (Criterion 43, MTS Systems, Eden Prairie, MN, USA). Briefly, the dorsal surface of the femur was supported by two rounded points spanning 6.4 mm with a loading plunger midway between the support points in contact with the ventral surface at the midshaft. Each bone was preloaded to 1 N in the ventral to dorsal direction before three‐point bend to fracture was completed with a crosshead speed of 0.1 mm/s and load‐displacement acquisition rate of 10 Hz. The main outputs include peak force (N) and stiffness (N/mm) calculated as the slope of the initial linear portion of the load‐displacement curve using two points acquired 0.3 seconds apart. Bone tissue‐level properties, ultimate stress (σ_U_, MPa), and estimated elastic modulus (E, GPa) were calculated using data obtained from whole‐bone mechanical testing and μCT analysis of cortical bone using previously published equations.^(^ ^18^, ^19^, ^20^ ^)^

### 2.4 Statistical analysis

Data were analyzed using IBM SPSS Statistics (version 26, IBM Corp, Armonk, NY, USA) and presented graphically using GraphPad Prism (version 8, GraphPad Software, La Jolla, CA, USA). Extreme values were identified using the formula: Q1 – (3 × IQR) > value > (3 × IQR) + Q3, where Q is quartile and IQR is the interquartile range of each outcome. A list of extreme values is included in Appendix S1. Some additional values were missing because of technical issues or user error, leaving a total of 11 to 14 samples per experimental group for each outcome assessed. After removal of extreme values, Shapiro–Wilk test for normality was used to identify non‐normally distributed data within each experimental group based on *p* < 0.05. Non‐normally distributed data were normalized using a natural log transformation before further analysis. A list of transformed outcomes is included in Appendix S1.

Initially, data were analyzed by two‐way analysis of variance (ANOVA) to probe for main effects of alcohol intake (dextrose control versus alcohol) and circadian status (normal versus disrupted) in each cohort and with the cohorts combined. A threshold of *p* < 0.05 was used to identify statistically significant differences between groups. A summary of the resulting probability values for main effects of alcohol and circadian status and interactions between alcohol and circadian status is included in Appendix S1. During the analysis, a possible cohort effect became apparent, so data were then analyzed by three‐way ANOVA to probe for main effects of cohort, alcohol intake, and circadian status and interactions between these variables.

Data are presented as non‐transformed group means and standard deviations in the text and in graphs. Individual non‐transformed data points are plotted and bars and error bars represent group means and standard deviations, respectively. Bar graphs are organized by cohort (cohort 1: winter born = gray bars on left; cohort 2: summer born = green bars on right), alcohol consumption (control diet = open bars; alcohol diet = striped bars), and circadian (circ) status (circ normal = open squares/circles; circ disrupted = black filled squares/circles). A threshold of *p* < 0.05 was used to identify statistically significant differences between groups. When *p* < 0.05, the effect size (ES) via partial eta squared is also presented. Effect sizes range from 0 to 1, with smaller effect sizes closer to 0 and larger effect sizes closer to 1. Significant main effects and interactions and their effect sizes are presented in tables adjacent to the graphs.

## 3 Results

### 3.1 Body mass was different between cohorts and was affected by alcohol intake

**Fig 1:**
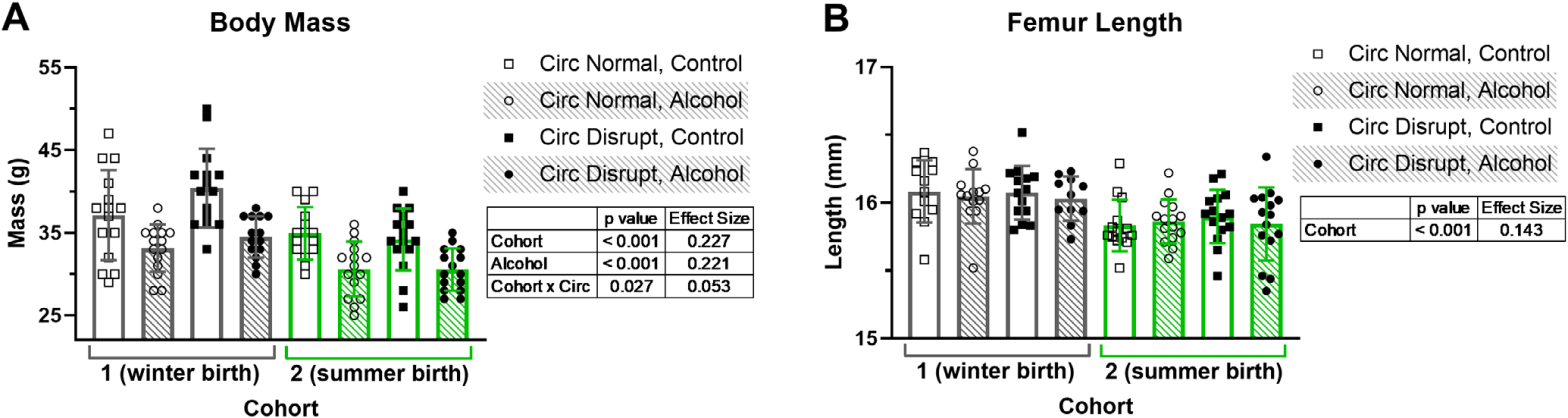
Body mass (*A*) and femur length (*B*) are plotted for two cohorts of mice. Data points represent individual mice and bars and error bars represent group mean and standard deviation, respectively. Three‐way ANOVA *p* values <0.05 and their associated effect sizes via the partial eta squared for each comparison are presented in tables adjacent to graphs. Circ = circadian.

Body mass was significantly affected by cohort (*p* < 0.001, ES = 0.227) and alcohol (*p* < 0.001, ES = 0.221, Fig. 1*A*). Mice from cohort 1 (winter born) tended to have higher body mass than cohort 2 (summer born), whereas mice who had alcohol in their diet had lower body mass than the control group, despite consuming statistically indistinguishable amounts of food (*p* > 0.05). Circadian rhythm disruption did not affect body mass.

### 3.2 Femur length was different between cohorts

Femur length was slightly higher in cohort 1 than cohort 2 (*p* < 0.001, ES = 0.143, Fig. 1*B*). Alcohol and circadian rhythm disruption did not affect femur length.

### 3.3 Cortical bone structure outcomes were different between cohorts and were not affected by alcohol intake or circadian disruption

**Fig 2:**
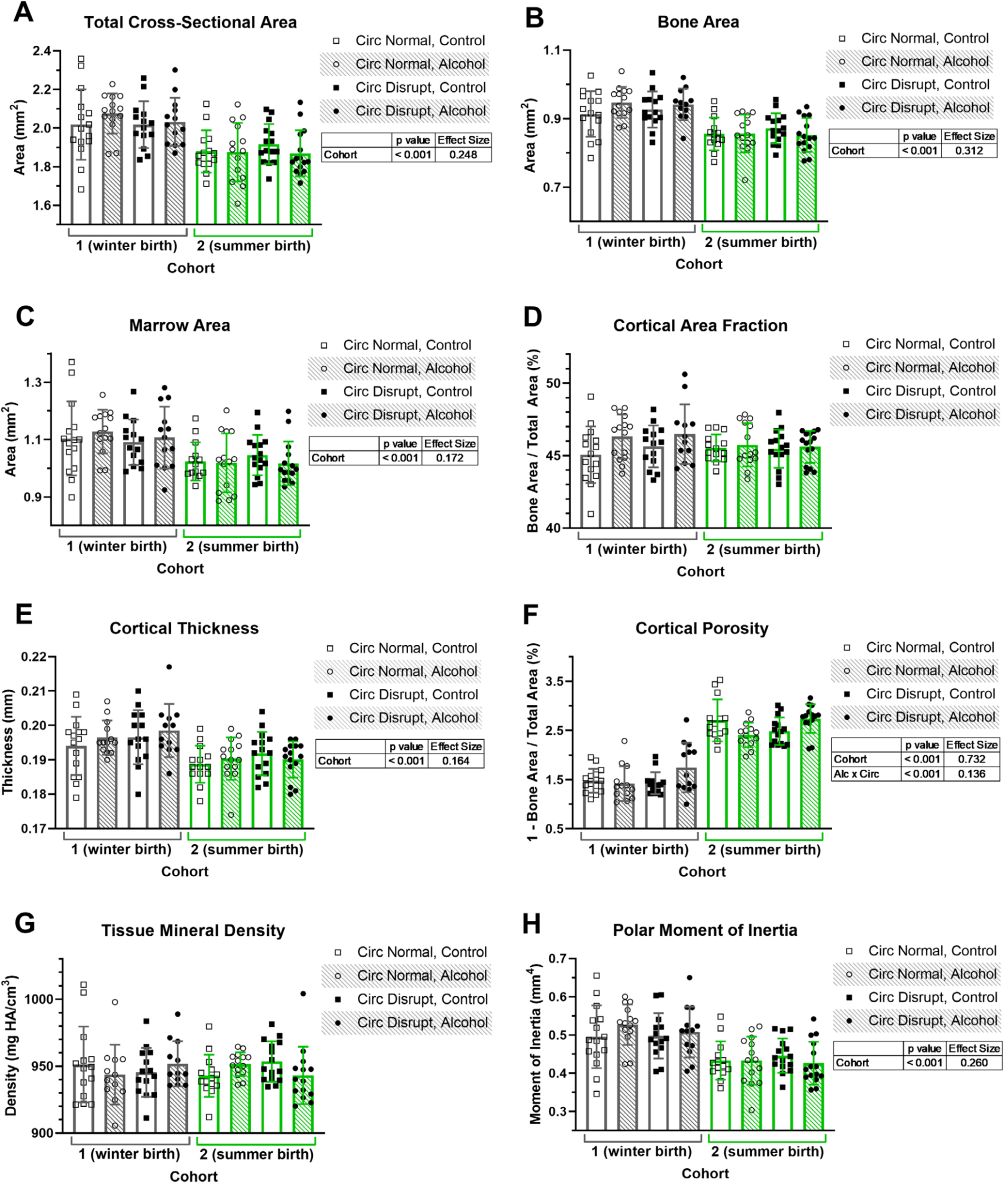
Cortical bone parameters derived from micro‐computed tomography analysis of the femoral midshaft are plotted (*A*–*H*) for two cohorts of mice. Data points represent individual mice and bars and error bars represent group means and standard deviations, respectively. Three‐way ANOVA *p* values <0.05 and their associated effect sizes via the partial eta squared for each comparison are presented in tables adjacent to graphs. Alc = alcohol; Circ = circadian.

Cortical bone structure, assessed by μCT at the femoral midshaft, was different in mice in cohort 1 compared with cohort 2 (Fig. 2). Specifically, total cross‐sectional area (*p* < 0.001, ES = 0.248, Fig. 2*A*), cortical bone area (*p* < 0.001, ES = 0.312, Fig. 2*B*), and marrow area (*p* < 0.001, ES = 0.172, Fig. 2*C*) were each larger in cohort 1 compared with cohort 2. Further, the cortex was thicker (*p* < 0.001, ES = 0.164, Fig. 2*E*) and less porous (*p* < 0.001, ES = 0.732, Fig. 2*F*) in cohort 1 than cohort 2. There was an interaction between alcohol consumption and circadian status for cortical porosity whereby alcohol‐fed, circadian‐disrupted mice had slightly higher porosity on average than the other groups (*p* < 0.001, ES = 0.136). The polar moment of inertia was also increased in cohort 1 compared with cohort 2 (*p* < 0.001, ES = 0.260, Fig. 2*H*). Cortical area fraction and tissue mineral density (TMD) were not affected by cohort, alcohol intake, or circadian disruption (Fig. 2*D*, *G*). Further, there were no effects of alcohol intake or circadian disruption alone on any cortical bone outcomes.

### 3.4 Trabecular bone structure outcomes were different between cohorts and were affected by alcohol intake and circadian status

**Fig 3:**
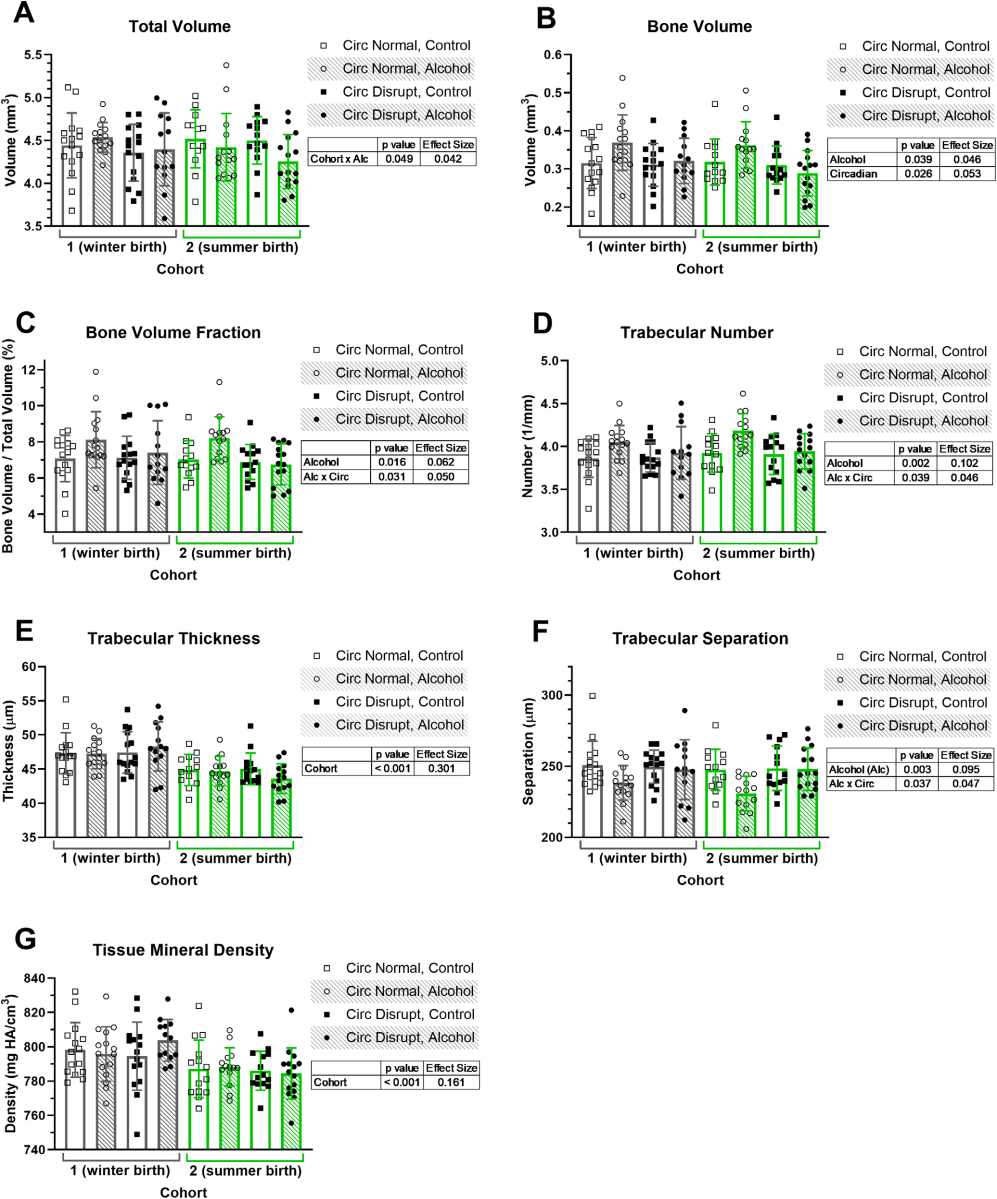
Trabecular bone parameters derived from micro‐computed tomography analysis of the distal femoral metaphysis are plotted (*A*–*G*) for two cohorts of mice. Data points represent individual mice and bars and error bars represent group means and standard deviations, respectively. Three‐way ANOVA *p* values <0.05 and their associated effect sizes via the partial eta squared for each comparison are presented in tables adjacent to graphs. Alc = alcohol; Circ = circadian.

Trabecular thickness and TMD were significantly different between cohorts, with increased thickness (*p* < 0.001, ES = 0.301) and TMD (*p* < 0.001, ES = 0.161) in cohort 1 compared with cohort 2 (Fig. 3*E*, *G*, respectively). No other trabecular bone outcomes were affected by cohort, except there was an interaction between cohort and alcohol intake for total volume (*p* = 0.049, ES = 0.042, Fig. 3*A*).

Mice that consumed alcohol and had a normal circadian rhythm had higher trabecular bone volume, bone volume fraction, and number, and lower trabecular separation than the other groups. Specifically, bone volume was affected by alcohol (*p* = 0.039, ES = 0.046), circadian status (*p* = 0.026, ES = 0.053), and the interaction of alcohol and circadian status (*p* = 0.028, ES = 0.052, Fig. 3*B*), whereas bone volume fraction was affected by alcohol (*p* = 0.016, ES = 0.062) and the interaction of alcohol and circadian status (*p* = 0.031, ES = 0.050, Fig. 3*C*). Alcohol intake and the interaction between alcohol intake and circadian status affected both trabecular number (alcohol: *p* = 0.002, ES = 0.102; alcohol × circadian disruption: *p* = 0.039, ES = 0.046, Fig. 3*D*) and separation (alcohol: *p* = 0.003, ES = 0.095; alcohol × circadian disruption: *p* = 0.037, ES = 0.047, Fig. 3*F*).

### 3.5 Whole‐bone mechanical properties were not affected by cohort, alcohol intake, or circadian disruption, but tissue‐level material properties were different between cohorts

**Fig 4:**
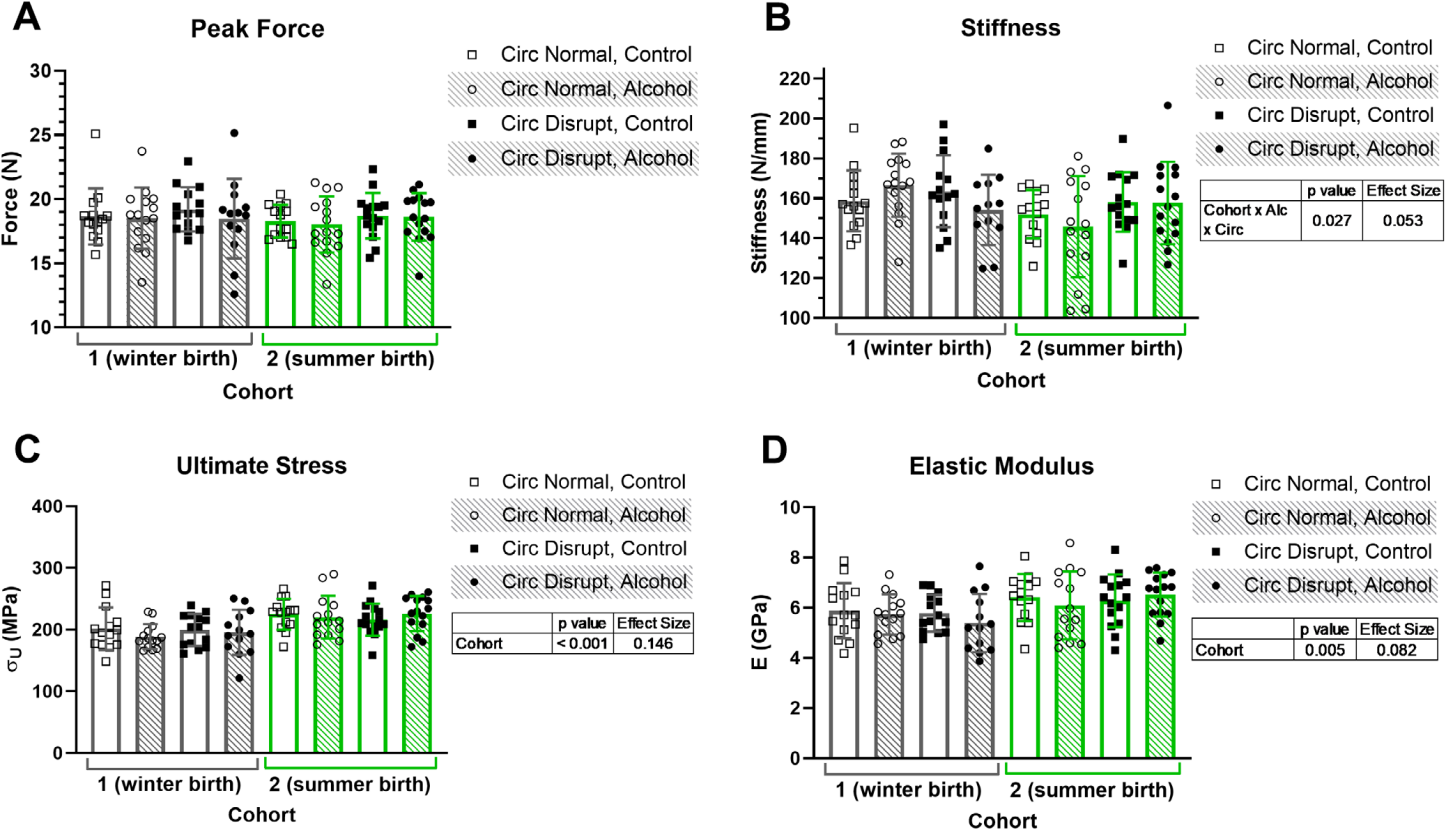
Mechanical properties, including peak force (*A*) and stiffness (*B*) derived from three‐point bend test of the femur, and calculated tissue‐level properties, including ultimate stress (*C*) and elastic modulus (*D*), are plotted for two cohorts of mice. Data points represent individual mice and bars and error bars represent group means and standard deviations, respectively. Three‐way ANOVA *p* values <0.05 and their associated effect sizes via the partial eta squared for each comparison are presented in tables adjacent to graphs. Alc = alcohol; Circ = circadian.

Peak force sustained during three‐point bend test of the femur was not affected by cohort, alcohol intake, or circadian disruption (Fig. 4*A*). Femoral stiffness was also not affected by cohort, alcohol intake, or circadian disruption alone, but there was an overall three‐way interaction between cohort, alcohol intake, and circadian status (*p* = 0.027, ES = 0.053, Fig. 4*B*). Ultimate stress and elastic modulus were calculated using data from μCT imaging and three‐point bend test of the femoral midshaft. Ultimate stress and elastic modulus were each lower in cohort 1 compared with cohort 2 (*p* < 0.001, ES = 0.146 and *p* = 0.005, ES = 0.082, Fig. 4*C*, *D*). There were no other significant effects on tissue‐level material properties.

## 4 Discussion

Our original intent was to assess the effects of environmental circadian disruption and chronic alcohol intake, alone and in combination, on bone structure and mechanical properties in mice. We found minimal impact of environmental circadian rhythm disruption on murine bone structure and mechanical properties, while chronic alcohol intake had significant effects on trabecular bone structure. Alcohol intake, particularly when circadian rhythm was normal, resulted in higher trabecular bone volume, but the beneficial effect of alcohol was blunted when circadian rhythm was disrupted. Interestingly, many outcomes were affected by cohort, including body mass, cortical bone (total area, bone area, marrow area, thickness, porosity, polar moment of inertia), trabecular bone (thickness and TMD), and calculated cortical bone tissue‐level material properties (ultimate stress and elastic modulus). All of these outcomes were higher in the cohort born in winter compared with the cohort born in summer, except cortical porosity, ultimate stress, and elastic modulus, which were lower in winter‐born mice.

Circadian disruption can impact bone^(^ ^5^, ^6^, ^7^, ^8^, ^9^ ^)^ and can be caused by shiftwork, which is modeled in the laboratory by exposing animals to aberrant light and dark cycles. Two previous studies assessed murine bone in response to this type of circadian disruption.^(^ ^10^, ^11^ ^)^ In the most recent study by Llabre and colleagues, light/dark cycles were altered on the same 3 days in a row each week for 22 weeks in a study that also exposed some mice to a high‐fat diet.^(^ ^11^ ^)^ Although the combination of circadian disruption and high‐fat diet readily induced hyperglycemia, effects of circadian disruption alone on bone structure and mechanical properties were less consistent. Circadian rhythm disruption (independent of diet) resulted in increased advanced glycation end products (AGEs) within bone tissue, suggesting an effect of circadian rhythm disruption on bone matrix properties. The second report utilized ApoE*3‐LeidenCETP female mice exposed to alterations in light/dark cycles for 15 weeks and found reduced serum bone turnover markers and cortical mineralization, but no differences in μCT‐based cortical bone structure or mechanical properties, and a nonsignificant increase in trabecular bone volume.^(^ ^10^ ^)^ Similarly in the present study, circadian disruption did not robustly affect bone structure or mechanical properties, even after 22 weeks of weekly disruption. It is possible that because mice had no other comorbidities and were not of advanced age, circadian rhythm disruption affected them less. Also, a longer duration of exposure to this type of circadian disruption or other patterns of aberrant light cycling may be required to observe effects on murine bone structure, but future studies will be needed to evaluate these possibilities.

Alcohol and bone have a complex relationship with effects ranging from beneficial to detrimental depending on age, frequency, and amount of alcohol consumed, along with other biological factors, including nutritional status and sleep schedule.^(^ ^12^ ^)^ In the current study, mice that chronically consumed alcohol (10 weeks) had higher trabecular bone volume than other groups, indicating protective and/or beneficial effects on bone, which is generally inconsistent with previous studies in rodent models.^(^ ^21^, ^22^ ^)^ It is possible that a longer duration of consumption or increased dose would be required to induce the expected negative effects of chronic alcohol on bone in mice^(^ ^23^ ^)^ or that an alternative method of alcohol delivery (eg, alcohol in water or alcohol gavage) would lead to different results. Bone remodeling is typically reduced by alcohol consumption, which may slow age‐related bone loss; however, alcohol also reduces bone formation.^(^ ^12^ ^)^ We observed higher trabecular bone volume in alcohol‐fed mice, which could have been caused by suppressed resorption due to reduced remodeling, assuming that the existing remodeling sites completed their bone formation process.^(^ ^24^ ^)^ Although circadian rhythm disruption did not blunt the protective effects of alcohol on cortical bone, the beneficial effects of alcohol on trabecular bone were not observed in circadian‐disrupted mice and cortical porosity was modestly higher when alcohol consumption was combined with circadian rhythm disruption. Taken together, these findings hint at the importance of maintaining a normal circadian rhythm when alcohol is consumed.

Although our observation of a cohort effect might simply reflect differences in animal cohorts due to breeding patterns or phytonutrient abundance rather than a consequence of birth season, several preclinical and clinical studies demonstrate the direct effects of birth season on bone. Delahunty and colleagues found sex‐ and bone compartment–specific seasonal differences in C57BL/6J mice.^(^ ^25^ ^)^ In their study, 16‐week‐old mice born in autumn were compared with mice born in spring. The study demonstrated that male mice born in autumn had higher body size and increased periosteal and endosteal circumferences at the femoral midshaft than mice born in spring. Similarly, body mass and many cortical bone outcomes in the present study were different between the cohorts. We acknowledge that factors thought to entrain seasonal rhythms in humans, such as length of day, temperature, and ultraviolet light exposure, are kept constant across seasons in the laboratory environment and cannot account for the differences observed in this study.^(^ ^3^ ^)^ However, taken together, these two studies suggest that there appear to be differences in murine bone structure that may be dependent upon season of birth. The environmental conditions that lead to these reported differences in bone structural parameters need to be further investigated.

Namgung and colleagues found that bone mineral content in human infants was higher in those born in winter compared with summer, even after controlling for race and sex.^(^ ^26^, ^27^ ^)^ Body weight and limb length have also been linked to season of birth in infants, with those born in autumn and winter having higher body weight and limb length, respectively, than infants born in other seasons.^(^ ^28^ ^)^ Further, Danish men and women born in the winter have a decreased risk for fracture later in life compared with those born in the summer.^(^ ^29^ ^)^ A decreased risk for fracture based on birth season is supported by other findings suggesting that birth weight and growth in early life predict bone mass, strength, and mineral content late in adulthood.^(^ ^30^, ^31^, ^32^ ^)^ These data are compelling and suggest that there are also differences in human bone structure based on season of birth.

Several mechanisms explaining seasonal effects have been suggested. For instance, maternal vitamin D, a molecule shown to vary by season and latitude,^(^ ^33^ ^)^ measured at approximately 18 weeks of gestation, was positively associated with offspring bone mineral content and density at 20 years of age.^(^ ^34^ ^)^ Another study reports that higher bone mineral content in infants born in winter was associated with decreased osteocalcin and vitamin D levels in serum.^(^ ^27^ ^)^ Serum melatonin and testosterone levels also vary seasonally in rats maintained on a constant light/dark schedule, with increased melatonin observed during winter and summer in both males and females and increased testosterone observed during the autumn in male rats,^(^ ^35^ ^)^ but the mechanisms contributing to this phenomenon are not yet clear. In the current study, cortical area and thickness, as well as trabecular thickness tended to be greater in male mice born in winter compared with summer. It is possible that molecules like osteocalcin, vitamin D, melatonin,^(^ ^36^ ^)^ and testosterone^(^ ^37^ ^)^ (known influencers of bone) exert direct or indirect effects on bone structure or mineralization during development that lead to the bone phenotypes we observed. Future studies will be needed to evaluate this phenomenon.

There are some limitations worth considering. This study included only adult, male mice. Age‐related bone loss is greater in female mice and they may be more sensitive to the effects of circadian disruption and alcohol.^(^ ^38^ ^)^ Future studies should examine serum or tissue level markers of bone turnover to better understand potential mechanisms of action. Another important limitation is the μCT scanning resolution that was used (10 μm), particularly when considering cortical porosity. This resolution is too coarse to accurately reflect all types of cortical porosity, such as lacunar‐canalicular osteocyte remodeling. The resolution used likely measures vascular pores, with some partial volume effects on results. Future studies should examine cortical porosity with finer resolution. Finally, we acknowledge the limitation that the breeding was performed by a commercial vendor.

In conclusion, cohort, or possibly season of birth, chronic alcohol consumption, and to a much lesser extent circadian rhythm disruption affect bone structure in normal laboratory male mice. These findings are important because most recognized drivers of seasonal variation, including day length and temperature, are kept constant in laboratory settings, suggesting some other internalization of seasonal time. Based on our results and prior studies, C57BL/6J mice may be a useful model organism to elucidate intrinsic mechanisms driving seasonal variations, which was also recently suggested by Reynolds and colleagues.^(^ ^39^ ^)^ Given the association of season of birth with bone density and fracture risk status later in life in humans, continued study of season of birth effects on bone is warranted.

Finally, from the perspective of scientific rigor, season of birth or cohort‐induced variation in experimental outcomes may degrade reproducibility of preclinical studies and should be considered in experimental design. Thus, we recommend consideration of season of birth and cohort in multi‐cohort studies and inclusion of date and/or season of birth in publications, which is not currently included in the ARRIVE guidelines for reporting animal research.^(^ ^40^ ^)^

## Disclosures

The authors have no conflicts of interest to declare.

5

### PEER REVIEW

The peer review history for this article is available at https://publons.com/publon/10.1002/jbm4.10591.

## Supporting information


**APPENDIX** **S1**. Supplemental Materials and Methods.

## References

[1] Hastings MH , Maywood ES , Brancaccio M . Generation of circadian rhythms in the suprachiasmatic nucleus. Nat Rev Neurosci. 2018;19(8):453‐469. 29934559 10.1038/s41583-018-0026-z

[2] Lincoln G . A brief history of circannual time. J Neuroendocrinol. 2019;31(3):e12694. 30739343 10.1111/jne.12694

[3] Golombek DA , Rosenstein RE . Physiology of circadian entrainment. Physiol Rev. 2010;90(3):1063‐1102. 20664079 10.1152/physrev.00009.2009

[4] Zvonic S , Ptitsyn AA , Kilroy G , et al. Circadian oscillation of gene expression in murine calvarial bone. J Bone Miner Res. 2007;22(3):357‐365. 17144790 10.1359/jbmr.061114

[5] Samsa WE , Vasanji A , Midura RJ , Kondratov RV . Deficiency of circadian clock protein BMAL1 in mice results in a low bone mass phenotype. Bone. 2016;84:194‐203. 26789548 10.1016/j.bone.2016.01.006 PMC4755907

[6] Takarada T , Xu C , Ochi H , et al. Bone resorption is regulated by circadian clock in osteoblasts. J Bone Miner Res. 2017;32(4):872‐881. 27925286 10.1002/jbmr.3053

[7] Swanson CM , Kohrt WM , Buxton OM , et al. The importance of the circadian system & sleep for bone health. Metabolism. 2018;84:28‐43. 29229227 10.1016/j.metabol.2017.12.002 PMC5994176

[8] Feskanich D , Hankinson SE , Schernhammer ES . Nightshift work and fracture risk: the Nurses' Health Study. Osteoporos Int. 2009;20(4):537‐542. 18766292 10.1007/s00198-008-0729-5 PMC2651998

[9] Quevedo I , Zuniga AM . Low bone mineral density in rotating‐shift workers. J Clin Densitom. 2010;13(4):467‐469. 21029978 10.1016/j.jocd.2010.07.004

[10] Schilperoort M , Bravenboer N , Lim J , et al. Circadian disruption by shifting the light‐dark cycle negatively affects bone health in mice. FASEB J. 2020;34(1):1052‐1064. 31914701 10.1096/fj.201901929R

[11] Llabre JE , Trujillo R , Sroga GE , Figueiro MG , Vashishth D . Circadian rhythm disruption with high‐fat diet impairs glycemic control and bone quality. FASEB J. 2021;35(9):e21786. 34411349 10.1096/fj.202100610RR PMC8534979

[12] Gaddini GW , Turner RT , Grant KA , Iwaniec UT . Alcohol: a simple nutrient with complex actions on bone in the adult skeleton. Alcohol Clin Exp Res. 2016;40(4):657‐671. 26971854 10.1111/acer.13000 PMC4918769

[13] Malik P , Gasser RW , Kemmler G , et al. Low bone mineral density and impaired bone metabolism in young alcoholic patients without liver cirrhosis: a cross‐sectional study. Alcohol Clin Exp Res. 2009;33(2):375‐381. 19053976 10.1111/j.1530-0277.2008.00847.x

[14] Santori C , Ceccanti M , Diacinti D , et al. Skeletal turnover, bone mineral density, and fractures in male chronic abusers of alcohol. J Endocrinol Invest. 2008;31(4):321‐326. 18475050 10.1007/BF03346365

[15] Summa KC , Voigt RM , Forsyth CB , et al. Disruption of the circadian clock in mice increases intestinal permeability and promotes alcohol‐induced hepatic pathology and inflammation. PLoS One. 2013;8(6):e67102. 23825629 10.1371/journal.pone.0067102 PMC3688973

[16] Nanji AA , Sadrzadeh SM , Dannenberg AJ . Liver microsomal fatty acid composition in ethanol‐fed rats: effect of different dietary fats and relationship to liver injury. Alcohol Clin Exp Res. 1994;18(4):1024‐1028. 7978082 10.1111/j.1530-0277.1994.tb00077.x

[17] Bouxsein ML , Boyd SK , Christiansen BA , Guldberg RE , Jepsen KJ , Müller R . Guidelines for assessment of bone microstructure in rodents using micro‐computed tomography. J Bone Miner Res. 2010;25(7):1468‐1486. 20533309 10.1002/jbmr.141

[18] Bhatia A , Albazzaz M , Espinoza Orías AA , et al. Overexpression of DMP1 accelerates mineralization and alters cortical bone biomechanical properties in vivo. J Mech Behav Biomed Mater. 2012;5:1‐8. 22100074 10.1016/j.jmbbm.2011.08.026 PMC3222863

[19] Schriefer JL , Robling AG , Warden SJ , et al. A comparison of mechanical properties derived from multiple skeletal sites in mice. J Biomech. 2005;38(3):467‐475. 15652544 10.1016/j.jbiomech.2004.04.020

[20] Jepsen KJ , Silva MJ , Vashishth D , et al. Establishing biomechanical mechanisms in mouse models: practical guidelines for systematically evaluating phenotypic changes in the diaphyses of long bones. J Bone Miner Res. 2015;30(6):951‐966. 25917136 10.1002/jbmr.2539 PMC4794979

[21] Wahl EC , Liu L , Perrien DS , et al. A novel mouse model for the study of the inhibitory effects of chronic ethanol exposure on direct bone formation. Alcohol. 2006;39(3):159‐167. 17127135 10.1016/j.alcohol.2006.08.004

[22] Johnson TL , Gaddini G , Branscum AJ , et al. Effects of chronic heavy alcohol consumption and endurance exercise on cancellous and cortical bone microarchitecture in adult male rats. Alcohol Clin Exp Res. 2014;38(5):1365‐1372. 24512198 10.1111/acer.12366 PMC4145819

[23] Hogan HA , Argueta F , Moe L , Nguyen LP , Sampson HW . Adult‐onset alcohol consumption induces osteopenia in female rats. Alcohol Clin Exp Res. 2001;25(5):746‐754. 11371724

[24] Jilka RL . The relevance of mouse models for investigating age‐related bone loss in humans. J Gerontol A Biol Sci Med Sci. 2013;68(10):1209‐1217. 23689830 10.1093/gerona/glt046 PMC3779631

[25] Delahunty KM , Horton LG , Coombs HF 3rd , et al. Gender‐ and compartment‐specific bone loss in C57BL/6J mice: correlation to season? J Clin Densitom. 2009;12(1):89‐94. 19195621 10.1016/j.jocd.2008.10.008 PMC3662003

[26] Namgung R , Mimouni F , Campaigne BN , Ho ML , Tsang RC . Low bone mineral content in summer‐born compared with winter‐born infants. J Pediatr Gastroenterol Nutr. 1992;15(3):285‐288. 1432466 10.1097/00005176-199210000-00009

[27] Namgung R , Tsang RC , Specker BL , Sierra RI , Ho ML . Low bone mineral content and high serum osteocalcin and 1,25‐dihydroxyvitamin D in summer‐ versus winter‐born newborn infants: an early fetal effect? J Pediatr Gastroenterol Nutr. 1994;19(2):220‐227. 7815245 10.1097/00005176-199408000-00013

[28] McGrath JJ , Keeping D , Saha S , et al. Seasonal fluctuations in birth weight and neonatal limb length; does prenatal vitamin D influence neonatal size and shape? Early Hum Dev. 2005;81(7):609‐618. 15972254 10.1016/j.earlhumdev.2005.03.013

[29] Abrahamsen B , Heitmann BL , Eiken PA . Season of birth and the risk of hip fracture in Danish men and women aged 65+. Front Endocrinol (Lausanne). 2012;3:2. 22645516 10.3389/fendo.2012.00002 PMC3355842

[30] Dennison EM , Syddall HE , Sayer AA , Gilbody HJ , Cooper C . Birth weight and weight at 1 year are independent determinants of bone mass in the seventh decade: the Hertfordshire cohort study. Pediatr Res. 2005;57(4):582‐586. 15695596 10.1203/01.PDR.0000155754.67821.CA

[31] Oliver H , Jameson KA , Sayer AA , Cooper C , Dennison EM , Hertfordshire Cohort Study Group . Growth in early life predicts bone strength in late adulthood: the Hertfordshire Cohort Study. Bone. 2007;41(3):400‐405. 17599849 10.1016/j.bone.2007.05.007 PMC2080691

[32] Baird J , Kurshid MA , Kim M , Harvey N , Dennison E , Cooper C . Does birthweight predict bone mass in adulthood? A systematic review and meta‐analysis. Osteoporos Int. 2011;22(5):1323‐1334. 20683711 10.1007/s00198-010-1344-9

[33] Christakos S , Dhawan P , Verstuyf A , Verlinden L , Carmeliet G . Vitamin D: metabolism, molecular mechanism of action, and pleiotropic effects. Physiol Rev. 2016;96(1):365‐408. 26681795 10.1152/physrev.00014.2015 PMC4839493

[34] Zhu K , Whitehouse AJO , Hart PH , et al. Maternal vitamin D status during pregnancy and bone mass in offspring at 20 years of age: a prospective cohort study. J Bone Miner Res. 2014;29(5):1088‐1095. 24189972 10.1002/jbmr.2138

[35] Kononenko N , Hnatiuk V . The study of the circannual relationship between the activity of the epiphysis and gonads in rats of different sex and age. Malays J Pathol. 2017;39(1):39‐45. 28413204

[36] Pandi‐Perumal SR , Srinivasan V , Maestroni GJM , Cardinali DP , Poeggeler B , Hardeland R . Melatonin: nature's most versatile biological signal? FEBS J. 2006;273(13):2813‐2838. 16817850 10.1111/j.1742-4658.2006.05322.x

[37] Ornoy A , Giron S , Aner R , Goldstein M , Boyan BD , Schwartz Z . Gender dependent effects of testosterone and 17 beta‐estradiol on bone growth and modelling in young mice. Bone Miner. 1994;24(1):43‐58. 8186733 10.1016/s0169-6009(08)80130-4

[38] Glatt V , Canalis E , Stadmeyer L , Bouxsein ML . Age‐related changes in trabecular architecture differ in female and male C57BL/6J mice. J Bone Miner Res. 2007;22(8):1197‐1207. 17488199 10.1359/jbmr.070507

[39] Reynolds JD , Case LK , Krementsov DN , Raza A , Bartiss R , Teuscher C . Modeling month‐season of birth as a risk factor in mouse models of chronic disease: from multiple sclerosis to autoimmune encephalomyelitis. FASEB J. 2017;31(6):2709‐2719. 28292961 10.1096/fj.201700062 PMC5434654

[40] Percie du Sert N , Hurst V , Ahluwalia A . The ARRIVE guidelines 2.0: updated guidelines for reporting animal research. PLoS Biol. 2020;18(7):e3000410. 32663219 10.1371/journal.pbio.3000410 PMC7360023

