# Supplementary material for: Alcohol and Circadian Disruption Minimally Impact Bone Properties in Two Cohorts of Male Mice While Between‐Cohort Differences Predominate: Association With Season of Birth?
Source: JBMR Plus. 2022 Jan 13;6(3):e10591. doi: 10.1002/jbm4.10591 (PMC8914150; doi:10.1002/jbm4.10591)

**Supplemental Information**

*1. Liquid diet for alcohol treatment*

The components of the liquid diet include: mineral mix, vitamin mix, choline bitartrate, d-L-methionine, lactalbumin, xanthan gum, dextrose (all obtained from Dyets, Inc., Bethlehem, PA), fish oil (from menhaden; from Sigma, St. Louis, MO) and Hershey’s Lite Chocolate syrup. The caloric composition of the diet was: 36% protein, 29% carbohydrate and 35% fat. At the time of tissue collection (which is a snapshot in time of chronic alcohol consumption), serum alcohol levels (measured by gas chromatography) in control-fed mice averaged 1.31 mg/dL (range 0-2.31) and alcohol-fed mice averaged 5.97 mg/dL (range 0-18.31).

*2. Tissue Collection*

Animals were sacrificed every 8 hours in each group to control for time-of-day effects (n = 5 per time of day per group). Kruskal-Wallis tests were used to assess bone outcomes by time of day within each group. Very few statistically significant differences were found within groups using p<0.05. Specifically, 8 of 168 comparisons (8 groups by 21 outcomes), or 4.8%, demonstrated a statistically significant difference. Given the negligible effect of time of day on bone structure and mechanical properties, it was not further considered in subsequent analyses.

*3. Statistical Outliers*

Circ = Circadian; Alc = Alcohol

- Length – 6 (all low): n=2 winter born, circ normal, control; n=1 winter born, circ normal, alcohol; n=1 winter born, circ disrupted, control; n=2 winter born, circ disrupted, alcohol
- Cortical Area Fraction – 1 (low) winter born, circ disrupted, alcohol
- Cortical Thickness – 1 (low) winter born, circ disrupted, alcohol
- Cortical TMD – 2 (high): n=1 winter born, circ normal, control; n=1 winter born, circ disrupted, control
- Trabecular Number – 1 (high) summer born, circ disrupted, alcohol
- Trabecular Thickness – 1 (high) summer born, circ disrupted, alcohol

*4. Non-Normally Distributed Outcomes Natural Log-Transformed for Statistical Analyses*

- Body Weight
- Femur Length
- Total Cross-Sectional Area (mid-shaft)
- Marrow Area (mid-shaft)
- Cortical Porosity (mid-shaft)
- Cortical Tissue Mineral Density (mid-shaft)
- Polar Moment of Inertia (mid-shaft)
- Trabecular Bone Volume Fraction (distal metaphysis)
- Trabecular Separation (distal metaphysis)

*5. Effects of alcohol intake and circadian disruption in each cohort and with cohorts combined*

Statistical assessment via 2-way analysis of variance (ANOVA) was conducted for each cohort and with cohorts combined. The results are summarized in text below and followed by a table presenting the resulting probability values. Probability values less than 0.05 are highlighted using bold text.

In cohort 1, the 2-way ANOVA showed that body mass and trabecular number were significantly affected by alcohol. Specifically, body mass was decreased in alcohol fed mice while trabecular number was increased in circadian normal, alcohol fed mice compared to the other groups. Cortical porosity was affected by the combination of alcohol intake and circadian disruption whereby porosity was slightly increased in alcohol treated mice when there was also a circadian rhythm disruption. There was also an interaction for stiffness where circadian normal, alcohol treated mice and circadian disrupted, control mice had increased stiffness compared to circadian normal, control and circadian disrupted, alcohol fed mice. However, there was a lot of overlap among individual points across the groups for stiffness.

In cohort 2, body mass and trabecular number and separation were affected by alcohol intake, while cortical porosity and tissue mineral density (TMD) were affected by the combination of circadian disruption and alcohol intake. Similar to cohort 1, body mass was decreased with alcohol intake, while trabecular number and separation were increased and decreased, respectively, in circadian normal, alcohol fed mice compared to the other groups, and cortical porosity was increased in circadian disrupted, alcohol fed mice compared to the other groups. Unlike in cohort 1, cortical tissue mineral density showed an interaction with slightly increased TMD in circadian normal, alcohol fed and circadian disrupted, control fed mice compared to circadian normal, control fed and circadian disrupted, alcohol fed mice, but there was a lot of overlap between the groups. Also, bone volume and bone volume fraction were slightly increased in circadian normal compared to circadian disrupted mice, however this increase was largely driven by increased bone volume in circadian normal, alcohol treated mice.

Analysis of the two cohorts combined demonstrated similar outcomes as the individual analyses. To summarize, body mass was reduced by alcohol intake while trabecular bone volume, bone volume fraction, and trabecular number were each increased and trabecular separation was decreased in circadian normal, alcohol fed mice compared to the other groups. Because we observed potential cohort effects when we plotted the data side-by-side, we also analyzed the outcomes by cohort. The results of the 2-way ANOVA are presented in the table below.


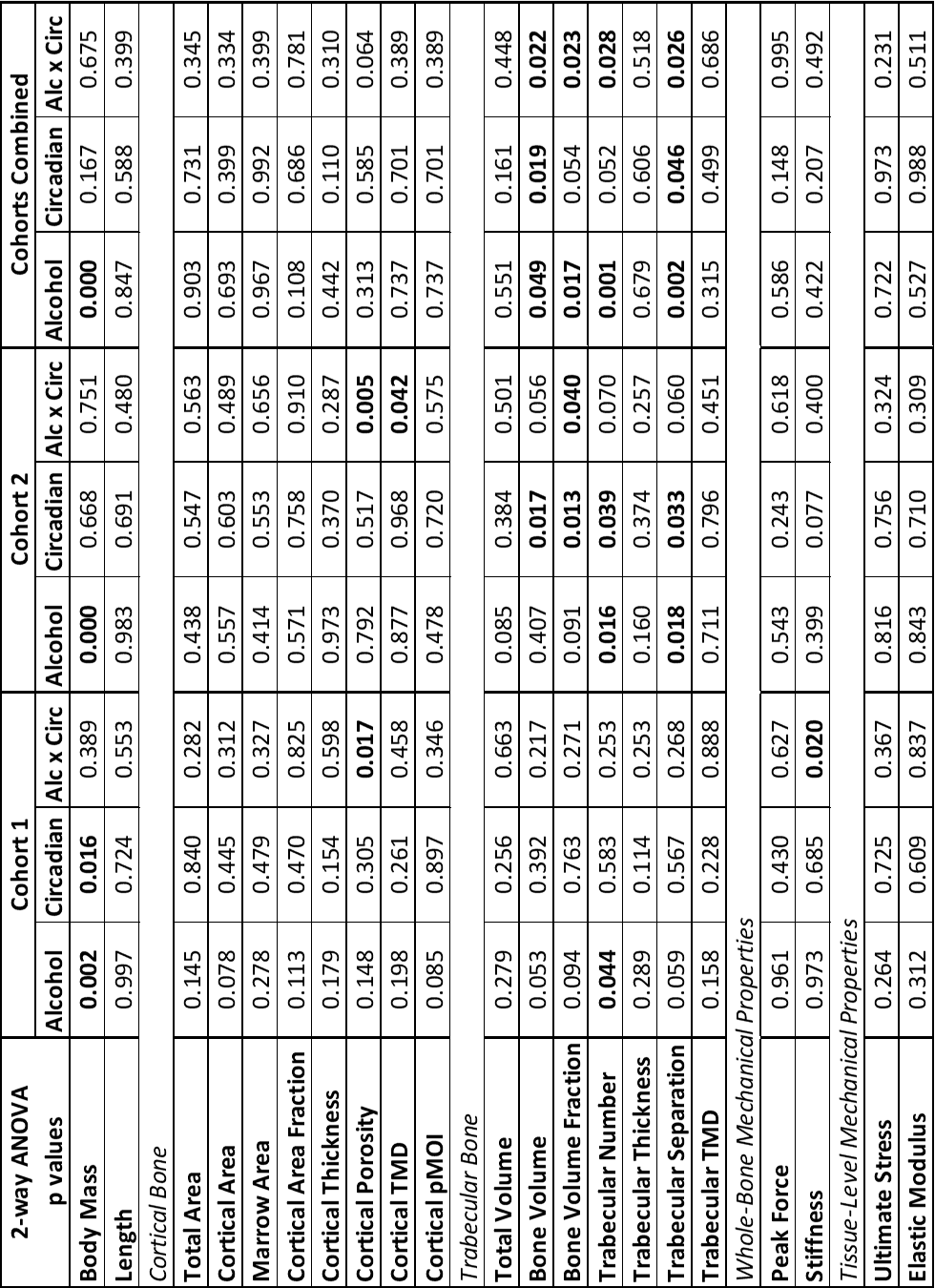

Supplement: Supplementary file 1 — APPENDIX S1. Supplemental Materials and Methods. [file JBM4-6-e10591-s001.doc]
